# Melamine‐Copolymerization Strategy Engineered Fluorinated Polyimides for Membrane‐Based Sour Natural Gas Separation

**DOI:** 10.1002/advs.202416109

**Published:** 2025-01-29

**Authors:** Yi Ren, Patrick T. Wright, Zhongyun Liu, Shijie Yang, Lu Lu, John Yang, Xuezhen Wang, Sheng Guo

**Affiliations:** ^1^ Aramco Americas Boston Research Center Cambridge MA 02139 USA; ^2^ School of Chemical and Biomolecular Engineering Georgia Institute of Technology Atlanta GA 30332 USA; ^3^ State Key Laboratory of Coordination Chemistry, MOE Key Laboratory of High‐Performance Polymer Materials & Technology, School of Chemistry and Chemical Engineering Nanjing University Nanjing 210023 China

**Keywords:** carbon dioxide, hydrogen sulfide, melamine, natural gas, polyimide membrane

## Abstract

Membrane‐based gas separation provides an energy‐efficient approach for the simultaneous CO_2_ and H_2_S removal from sour natural gas. The fluorinated polyimide (PI) membranes exhibited a promising balance between permeability and permselectivity for sour natural gas separation. To further improve the separation efficiency of fluorinated PI membranes, a melamine‐copolymerization synthetic approach is devised that aims to incorporate melamine motifs with high sour gas affinity into the structure of the PI membranes. The fluorinated copolyimide membranes that are structurally engineered exhibited excellent solution‐processability and enhanced sweet‐mixed gas selectivity compared to their original PI membranes. Additionally, under a five‐component sour mixed‐gas feed, these melamine‐copolymerized fluorinated PI membranes provided superior combined H_2_S and CO_2_ removal efficiency in comparison to conventional glassy polymer membranes. The melamine‐copolymerization strategy provides an easily operable and generally effective approach to developing performance‐enhancing PI membranes for sour natural gas separation.

## Graphical abstract

A synthetically efficient molecular engineering strategy has been demonstrated for fabricating high‐efficiency sour natural gas separation membranes. The incorporation of melamine‐analogues with high sour gas (CO_2_ and H_2_S) affinity endows the functionalized polyimide membranes with excellent solution‐processability and good sour gas selectivity. This strategy is generalized for multiple polyimide structures to develop membranes with enhanced performance for sour natural purification.

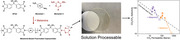

## 1 Introduction

Natural gas (NG), as the cleanest‐burning hydrocarbon source, plays a crucial role in fulfilling continuously increasing global energy demand.^[^ ^1^ ^]^ Even though the raw natural gas consists mostly of methane, it also contains significant amounts of undesired acid gas, including carbon dioxide (CO_2_) and hydrogen sulfide (H_2_S).^[^ ^2^ ^]^ These harmful gases decrease the heating value of natural gas, cause damage to transportation pipelines and equipment, and pose a potential risk owing to the high toxicity of H_2_S.^[^ ^3^ ^]^ In order to meet the NG sales specifications, liquid amine scrubbing has been used to remove the acid gases in raw NG before NG transportation. However, amine scrubbing incurs a significant operational expense due to high regeneration heat and requires the use of intricate processing equipment and substantial quantities of chemical solvents.^[^ ^4^ ^]^

**Figure 1:**
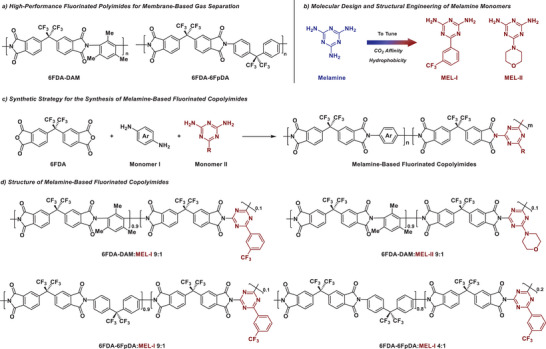
a) High‐performance fluorinated polyimides for membrane‐based gas separation. b) Molecular design and structural engineering of melamine monomers. c) Synthetic strategy for the synthesis of melamine‐based fluorinated copolyimides. d) Structure of melamine‐based fluorine‐rich copolyimides.

Membrane‐based gas separation technology, combining the advantages of high energy efficiency, excellent scalability, and small footprint, provides a promising approach for sweetening sour natural gas.^[^ ^3^, ^5^ ^]^ Unfortunately, the separation process achieved by the standard industrial polymeric membranes displays low performance and is unable to compete with the conventional technologies for the purification of raw NG.^[^ ^5^, ^6^ ^]^ To achieve high‐efficiency separation technique for the simultaneous removal of CO_2_ and H_2_S, it is imperative that the polymer membranes possess exceptional separation performance in terms of CO_2_/CH_4_, H_2_S/CH_4_ selectivity, and excellent plasticization resistance.^[^ ^2^, ^7^ ^]^ Fluorinated polyimide membranes, such as 6FDA‐DAM and 6FDA‐6FpDA (**Figure** **1**a), exhibited high H_2_S and CO_2_ co‐removal efficiency under high pressure sour natural gas feed.^[^ ^2^, ^8^ ^]^ The bulky hexafluoroisopropylidene ‐C(CF_3_)_2_‐ groups can provide the polymer membranes with high free volume and good solution‐processability.^[^ ^8^, ^9^ ^]^ To further improve the performance of these fluorinated homo‐polyimides, 6FDA‐DAM: DABA and 6FDA‐6FpDA: Durene were prepared via a copolymerization strategy. The performance of these copolyimide membranes was reported to be superior to their corresponding homopolymer membranes for sour mixed‐gas separation.^[^ ^2^, ^5^, ^8^, ^10^ ^]^ Therefore, the copolymerization strategy by employing a comonomer with specific functional moieties provides an effective way to design and engineer advanced membrane materials for sour natural gas separation.

Melamine (MEL) (Figure 1b), which has rich nitrogen content and strong H_2_S and CO_2_‐binding affinity,^[^ ^11^ ^]^ is an ideal comonomer for constructing the copolyimide membranes with potentially enhanced sour gas (CO_2_ and H_2_S) separation performance. Melamine is often employed as a rigid building block to construct 3D porous organic polymers for adsorption‐based sour gas separation.^[^ ^12^ ^]^ There are exceedingly few examples of MEL‐based polymers for membrane‐based separation. Although MEL‐based covalent organic framework mixed matrix membranes and melamine‐crosslinked poly(ethylene glycol) membranes for sour gas separation have been reported,^[^ ^11^, ^13^ ^]^ solution‐processable MEL‐based pure polymers have never been previously reported for membrane‐based sour gas separation. The membranes with solution processability and ease of manufacturing that can be used “as is”, are of great value to the practical application of gas separation.^[^ ^14^ ^]^ However, it remains extremely challenging to develop solution‐processable MEL‐based polymer materials. First, the polycondensation of melamine having three amino groups produces an insoluble polymer with a high degree of crosslinking, compromising the excellent processability of the polymer material. Second, MEL‐based polymers have high hydrophilicity that results in low solubility in commonly used organic solvents, which reduces the synthesis efficiency.^[^ ^12^, ^15^ ^]^

To develop solution‐processable MEL‐based copolyimides, the melamine‐analogue comonomers were designed and synthesized (Figure 1b). These melamine‐analogues are highly synthetically flexible and contribute to several advantages for copolyimide membranes. First, due to the nature of diamine monomers, the MEL comonomers designed can be used for preparing linear MEL‐based polymers with excellent processability and no crosslinking. Furthermore, the incorporation of fluorinated aryl groups and aliphatic cyclic amines in melamine monomers can not only enhance the hydrophobicity of MEL‐based polymers, thereby rendering them soluble in organic solvents,^[^ ^16^ ^]^ but also regulate the CO_2_ affinity of the polymer membranes (Figure 1b).^[^ ^8^, ^17^ ^]^ Herein, we developed a copolymerization strategy for incorporating the well‐designed MEL structures into the skeleton of the fluorinated polyimides to provide performance‐enhancing membrane materials (Figure 1c).

## 2 Results and Discussion

Four MEL‐based fluorinated copolyimides were obtained by this synthetic strategy. The 6FDA‐DAM:MEL‐I 9:1 and 6FDA‐DAM: MEL‐II 9:1 were prepared using MEL‐I and MEL‐II as comonomers, respectively. To demonstrate the general effectiveness of this synthetic strategy, 6FDA‐DAM:MEL‐I 9:1 and 6FDA‐6FpDA:MEL‐I 9:1 was synthesized using different dianhydride and diamine combinations. Additionally, 6FDA‐6FpDA:MEL‐I 4:1 with a higher content of melamine than 6FDA‐6FpDA:MEL‐I 9:1 was prepared (Figure 1d) to investigate the effect of the content of melamine structure on the performance of MEL‐based copolyimide membranes. These copolyimides exhibit high thermal stability and excellent solution processability which allows them to be prepared as robust films via solution casting process, and they can be used directly without post‐treatments (such as crosslinking) for the membrane‐based testing (Figures S1–S4, Supporting Information).

**Figure 2:**
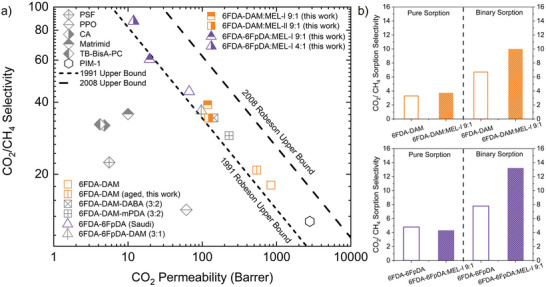
a) Robeson upper bound plots for four novel melamine‐based copolyimide for CO_2_/CH_4_ pure gas separation (Table S2, Supporting Information). The dashed lines represent 2008 and 1991 upper bounds.^[^ ^20^ ^]^ The original polymers 6FDA‐DAM and 6FDA‐6FpDA, along with other commonly co‐polymerized polymers and commercially available polymers are shown.^[^ ^21^ ^]^ b) Top: Pure CO_2_/CH_4_ sorption selectivity data and binary sorption prediction for 6FDA‐DAM versus 6FDA‐DAM:MEL‐I 9:1. Bottom: Pure CO_2_/CH_4_ sorption selectivity data and binary sorption prediction for 6FDA‐6FpDA versus 6FDA‐6FpDA:MEL‐I 9:1.

Pure CO_2_ and CH_4_ permeabilities of these four melamine‐based fluorinated copolymer membranes were measured at 25 °C (**Figure** **2**a). It was observed that a trend of increased selectivity with decreased permeability holds true for all four melamine‐functionalized polymer membranes compared to their parent membranes (shown in orange squares and purple triangles). Comparing two melamine based 6FDA‐DAM co‐polymer membranes (6FDA‐DAM:MEL‐I 9:1 and 6FDA‐DAM:MEL‐II 9:1, orange squares), different melamine structure resulted in different separation performances, with MEL‐I in the 6FDA‐DAM:MEL‐I 9:1 considered as more effective since both polymer membranes exhibit similar permeability while 6FDA‐DAM:MEL‐I 9:1 was more selective (ideal selectivity of 39 vs34). Since MEL‐I was identified as the superior melamine monomer, MEL‐I was used for 6FDA‐6FpDA co‐polymerization. The comparison of 6FDA‐DAM:MEL‐I 9:1 and 6FDA‐6FpDA:MEL‐I 9:1 revealed that the parent polymer membrane performance would affect its functionalized version, with 6FDA‐DAM:MEL‐I 9:1 being more permeable and less selective due to the intrinsic nature of pristine 6FDA‐DAM.^[^ ^2b^ ^]^ Finally, to determine the effect of melamine concentration, the comparison of 6FDA‐6FpDA:MEL‐I 9:1 and 6FpDA:MEL‐I 4:1 (purple triangles) revealed that 6FDA‐6FpDA:MEL‐I 4:1 exhibited higher permselectivity (87 versus 60) and lower permeability (11 versus 20 Barrer), indicating that an increase in melamine concentration in the polyimide backbone can effectively enhance selectivity while sacrificing permeability. The upper bound analysis showed that this melamine strategy was generally effective for enhancing the performance of the glassy polyimide membranes. Due to the high performance of 6FDA‐DAM:MEL‐I 9:1 and 6FDA‐6FpDA:MEL‐I 9:1, these two polymer membranes were further investigated in more detail for CO_2_ sorption.

To fundamentally understand the effect of incorporating the melamine structure on CO_2_ and CH_4_ sorption in the fluorinated polyimide membranes, the sorption coefficient of CO_2_ and CH_4_ was measured using a pressure decay sorption setup (Figure S5, Supporting Information). Four polymer membranes were measured for CO_2_ and CH_4_ under 100 psi, 6FDA‐DAM:MEL‐I 9:1 with pristine 6FDA‐DAM and 6FDA‐6FpDA:MEL‐I 9:1 with pristine 6FDA‐6FpDA. It was observed that the CO_2_/CH_4_ pure sorption selectivity of the melamine functionalized polymer membrane was similar to that of the parent one (Figure 2b, left parts and Table S3, Supporting Information). However, through a mixed gas dual mode sorption model developed by Koros,^[^ ^18^ ^]^ CO_2_ and CH_4_ sorption isotherms (Figure S6, Supporting Information) of the four membranes under 20/80 binary CO_2_/CH_4_ mixture were predicted using the dual mode sorption parameters in Table S4 (Supporting Information). With those information, CO_2_/CH_4_ sorption selectivity under 20/80 binary CO_2_/CH_4_ mixture was calculated and the melamine functionalized polymer membranes showed a significantly higher sorption selectivity in 20/80 binary CO_2_/CH_4_ mixture than the parent polymers (Figure 2b, right parts) owing to the higher CO_2_ Langmuir affinity constant, resulting in strong competitive sorption favoring CO_2_ over CH_4_,^[^ ^5^, ^19^ ^]^ supporting the design strategy of this work.

**Figure 3:**
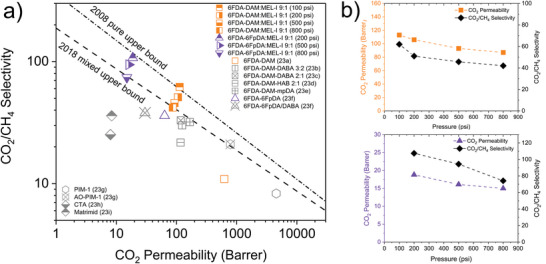
a) CO_2_/CH_4_ separation performance comparison of melamine‐based copolyimide membranes under sweet mixed‐gas (20/80 mol% CO_2_/CH_4_) with pressure of 100, 200, 500, and 800 psi at 25 °C (Table S5, Supporting Information). 2008 pure gas upper bound and 2018 mixed gas upper bound were shown for comparison.^[^ ^20^, ^22^ ^]^ Other commonly used co‐polymers are also shown for reference.^[^ ^23^ ^]^ 6FDA‐DAM:MEL‐1 9:1 (half‐filled orange square), 6FDA‐6FpDA‐DAM:MEL‐1 9:1 (half‐filled purple triangle); other glass polymer membrane reported in literature: 6FDA‐DAM (hollow orange square), 6FAM‐DAM copolymer family (grey square), 6FDA‐6FpDA (hollow purple triangle), 6FDA‐6FpDA:DABA (grey triangle), PIM‐1 (hollow hexagon), AO‐PIM‐1 (crossed hexagon), CTA (bottom filled diamond), Matrimid (top filled diamond). b) Top: Permeability and selectivity of 6FDA‐DAM: MEL‐1 9:1 using (20 mol%/80 mol%) of CO_2_/CH_4_ under 100, 200, 500, 800 psi. Bottom: Permeability and selectivity of 6FDA‐6FpDA: MEL‐1 9:1 using (20 mol%/80 mol%) of CO_2_/CH_4_ under 200, 500, 800 psi. [Correction added on 31 January 2025, after first online publication: figure labels a) and b) added.]

With a promising CO_2_/CH_4_ binary sorption selectivity prediction, sweet‐mixed binary gas permeation testing of 6FDA‐DAM: MEL‐I 9:1 and 6FDA‐6FpDA‐MEL‐I 9:1 was carried out for evaluating their potentials in the practical mixture application. Under sweet mixed‐gas feed (20/80 mol% of CO_2_/CH_4_) with pressure from 100 to 800 psi at 25 ^°^C, both 6FDA‐DAM: MEL‐I 9:1 and 6FDA‐6FpDA‐MEL‐I 9:1 showed outstanding performance (**Figure** **3**a), exceeding the 2018 mixed upper bound, with slightly lower selectivity and permeability as the pressure increased.

Figure 3b shows the CO_2_/CH_4_ binary gas separation performance of both 6FDA‐DAM: MEL‐I 9:1 and 6FDA‐6FpDA: MEL‐I 9:1 as pressure increased. As expected from the pure gas performance, 6FDA‐6FpDA:MEL‐I 9:1 exhibited higher CO_2_/CH_4_ selectivity and lower permeability than 6FDA‐DAM:MEL‐I 9:1. It was worth noting that in binary mixture separation, both polymer membranes had higher CO_2_/CH_4_ selectivity compared to pure gas permeation. Specifically, the CO_2_/CH_4_ mixed gas selectivity of 6FDA‐DAM:MEL‐I 9:1 was 46 under 500 psi (100 psi of CO_2_ partial pressure), which is higher than its ideal gas selectivity of 39.0 under 100 psi. Additionally, the CO_2_/CH_4_ mixed gas selectivity of 6FDA‐6FpDA‐MEL‐I was 94, which surpassed its ideal gas selectivity of 60 under the same 100 psi of CO_2_ partial pressure. Their higher selectivity under binary mixture gas was predicted by the dual mode sorption model in Figure 2b, demonstrating how the incorporation of melamine monomers into the polyimide backbone could achieve enhanced performance in binary mixtures for natural gas purification. Both polymers experienced decrease in selectivity and permeability as pressure increased from 100 to 800 psi. The CO_2_ permeability decreased in the pressure range for both polymers, suggesting that the incorporation of melamine monomers into PI backbone can potentially preserve polymer chain mobility and reduce plasticization effect. The decrease in selectivity could be explained by decreased sorption selectivity from gas sorption isotherms of CO_2_ and CH_4_ as pressure increased. With suppressed plasticization, dual model effect from gas sorption isotherm resulted in lower permeability due to saturation of Langmuir pores with increased pressure.^[^ ^24^ ^]^ The parent polymers 6FDA‐DAM and 6FDA‐6FpDA, along with other commercially available polymers, failed to exceed the upper bound, highlighting the importance of melamine incorporation in this application.

**Figure 4:**
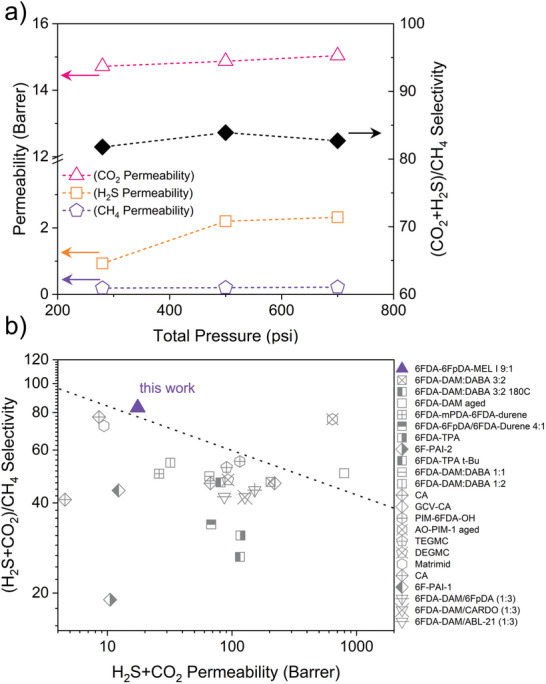
a) Separation performance of 6FDA‐6FpDA‐MEL‐I 9:1 with a feed mixture of 10 mol% CO_2_, 20 mol% H_2_S, 57 mol% CH_4_, 3 mol% C_2_H_6_, and 10 mol% N_2_ under 25 °C, with increasing pressure from 280 to 700 psi. b) Combined acid gas separation performance of melamine‐based copolyimide membranes and other glassy polymer membranes reported in literature. (Table S7, Supporting Information). Dashed line qualitatively defines state of the art performance from glassy polymer membranes.

Finally, due to the outstanding performance of 6FDA‐6FpDA‐MEL‐I 9:1, which showed the higher CO_2_/CH_4_ selectivity of 107 under 200 psi for the binary mixture, and a reasonable permeability of 18 Barrer, the 6FDA‐6FpDA:MEL‐I 9:1 membrane was challenged with a realistic and more aggressive five‐component sour natural gas mixture^[^ ^5^, ^10^ ^]^ containing 10 mol% CO_2_, 20 mol% H_2_S, 57 mol% CH_4_, 3 mol% C_2_H_6_ and 10 mol% N_2_ under industrial operation condition at 25 °C with a total pressure from 280 to 700 psi. The overall performance of this membrane was shown in **Figure** **4**a (Table S6, Supporting Information). Under 700 psi, (210 psi of CO_2_/H_2_S partial pressure), 6FDA‐6FpDA‐MEL‐I 9:1 exhibited a competitive CO_2_/CH_4_ selectivity of ≈72, comparable to that observed in binary mixture, surpassing that observed with pure gases, and a (CO_2_+H_2_S)/CH_4_ selectivity of 83. As pressure increases, the CO_2_ and H_2_S permeability increase slightly due to the plasticization effect under elevated pressure.^[^ ^25^ ^]^ It is worth noting that since H_2_S serves as a stronger plasticizer than CO_2_, some level of plasticization was observed as opposed to the binary mixed gas scenario with only CO_2_ present in the feed. However, plasticization induced by the introduction of 20 mol% H_2_S in the feed only resulted in a minor increase in CH_4_ permeability due to the strong competition effect of CO_2_ and H_2_S over CH_4_. This transport mechanism enabled balanced plasticization/competition effect in sour natural gas separation,^[^ ^9^, ^26^ ^]^ with well‐maintained (CO_2_+H_2_S)/CH_4_ selectivity in the range of 82–84 as pressure increased from 280 to 700 psi. The consistent selectivity with slightly increasing acid gas permeability suggests that the melamine incorporation design can mitigate and even utilize membrane plasticization under high pressure sour natural gas environments where H_2_S is present. Figure 4b compares 6FDA‐6FpDA‐MEL‐I 9:1 with other state of the art glassy polymer membranes reported in literature. From the comparison the newly designed 6FDA‐6FpDA‐MEL‐I 9:1 has the highest acid gas removal efficiency with decent permeability. This suggests that the melamine‐incorporated 6FDA‐6FpDA structure is particularly suitable for industrial CO_2_ removal from natural gas. Although it is still challenging to achieve ppm level purification specifications of H_2_S removal with high H_2_S concentration feed, the integration of high performing membranes such as 6FDA‐6FpDA‐MEL‐I 9:1 presented here, into other separation processes or using multi‐stage membrane separation system can potentially achieve highly efficient separation processes with lower cost, carbon emission and smaller footprint.

## 3 Conclusion

In conclusion, a series of solution‐processable melamine incorporated fluorinated polyimide co‐polymers were prepared for membrane‐based sour natural gas purification. Although this melamine incorporation technique was successful for two major polyimide structures explored here, the general chemistry could be potentially applied to other polyimide structures given the optimal experimental conditions are determined. Preliminary pure gas screening measurement showed promising results comparing melamine incorporated polymers with original parent polymers. Binary mixture test with CO_2_ and CH_4_ revealed the selectivity enhancement from competitive sorption due to the incorporation of melamine. Finally, in a five‐component sour natural gas mixture, 6FDA‐6FpDA‐MEL‐I 9:1 could successfully resist plasticization and maintain high selectivity with the presence of 20 mol% H_2_S. This material design offers an alternative approach to developing solution‐processable glassy polymer membranes with high separation performance. Pure polymers with high processability, such as the MEL‐PI designed in this work, could be potentially transferred to membrane morphologies such as asymmetric or composite hollow fibers that are industrially relevant in clean energy applications.

## Conflict of Interest

The authors declare the following competing financial interest(s): The matter in this study is pending a patent titled “Melamine‐containing co‐polyimide membranes for natural gas separation” (Application No.: 19/013466) assigned to Saudi Arabian Oil Co.

## Supporting information



Supporting Information

## Data Availability

The data that support the findings of this study are available in the supplementary material of this article.
