# Supplementary material for: Melamine‐Copolymerization Strategy Engineered Fluorinated Polyimides for Membrane‐Based Sour Natural Gas Separation
Source: Adv Sci (Weinh). 2025 Jan 29;12(11):2416109. doi: 10.1002/advs.202416109 (PMC11923909; doi:10.1002/advs.202416109)
Supplement: Supplementary file 1 — Supporting Information [file ADVS-12-2416109-s001.docx]

Supplementary Information for

Melamine-Copolymerization Strategy Engineered Fluorinated Polyimides for Membrane-Based Sour Natural Gas Separation

Yi Ren, Patrick T. Wright, Zhongyun Liu, Shijie Yang, Lu Lu, John Yang*, Xuezhen Wang*, Sheng Guo*

# Contents

Contents 2

1. General Information for Materials and Analytical Methods 3

2. Synthesis of Polymers 5

3. Characterization Data for Polymers 6

3.1 Characterization Data for Melamine-Based Copolyimides 6

3.2 Characterization Data for Monomers 10

4. NMR Spectra 11

5. Gel Permeation Chromatography 21

6. Thermogravimetric Analysis and Differential Scanning Calorimetry 23

7. Membrane Fabrication 25

8. Membrane Permeation Testing 26

9. Pure Gas Permeability Data 28

10. Sorption Analysis 28

11. Binary Gas Permeability Data 31

12. Business case sour natural gas with five components 32

13. Sour gas mixed gas separation performance 32

14. References 34

# General Information for Materials and Analytical Methods

Unless noted, all chemicals were obtained from commercial sources, stored at room temperature, and used as received. All polycondensation reactions were carried out under an atmosphere of nitrogen using standard Schlenk techniques. Anhydrous DMF (N, N-dimethylformamide) was purchased from Sigma-Aldrich in Sure-Seal^TM^ bottles. Other reagents were purchased from chemical suppliers (Sigma-Aldrich, Ambeed, TCI America, Acros). Products were purified by flash chromatography using SiliCycle SiliaFlashP60 (230–400 mesh) silica gel with the aid of a Biotage Automated Flash Chromatography System.

Nuclear magnetic resonance (NMR): ^1^H, ^13^C, and ^19^F NMR spectra were recorded on a Bruker Avance-500 MHz spectrometer. ^1^H and ^13^C spectra were calibrated using residual solvent as an internal reference (CHCl_3_: δ 7.26 ppm and δ 77.36 ppm, respectively; DMSO: 2.50 ppm and 39.52 ppm, respectively). Broadband ^1^H decoupling was used during the collection of ^13^C and ^19^F NMR spectra. The following abbreviations were used to denote multiplicities: s = singlet, bs = broad singlet, d = doublet, t = triplet, q = quartet, p = pentet, and m = multiplet.

Gel permeation chromatography (GPC): Measurements were carried out in HPLC-grade tetrahydrofuran using an Agilent 1260 Infinity system with variable wavelength diode array (254, 450m and 530 nm) and refractive index detectors, guard column (Agilent PLgel; 5 µm; 50 x 7.5 mm), and three analytical columns (Agilent PLgel; 5µm; 300 x 7.5 mm; 105, 104, and 103 Å pore sizes). The instrument was calibrated with narrow-dispersity polystyrene standards between 1.7 and 3150 kg mol^−1^. All runs were performed at 1.0 mL min^−1^ flow rate and 35 ºC. Molecular weight values were calculated using ChemStation GPC Data Analysis Software (Rev. B.01.01) based on the refractive index signal.

Thermogravimetric analysis (TGA): TGA measurements were carried out under nitrogen atmosphere (AirGas, ultra-high purity grade) using a TGA1- 0280 from TA Instruments. The ramp speed was 10 °C min^−1^, and operation were performed from room temperature to 750 °C. The degradation temperature (T_d_) of a polymer is the temperature at which the polymer loses 10% of its initial mass. Thermal transitions were determined by differential scanning calorimetry (DSC) using a DSC1-0303 from TA instruments with powdered samples (5-8 mg) sealed in aluminum pans.

Fourier-transform infrared (FT-IR) spectroscopy: All IR spectra were obtained on a Thermo Scientific Nicolet iS50 spectrometer (iD5 ATR, diamond).

Gas permeation experiments were performed in a custom built constant-volume, variable-pressure system described in details in Figure S4. All gas used for measurement were ultra-high purity grade from Air Gas. For mixture gas, Shimadzu gas chromatograph (GC-2014) was used to measure permeate composition.

# Synthesis of Polymers

***General Procedure*:** An oven-dried Schlenk flask (purchased from Synthware^TM^, modified, with PTFE stopcock, F909100, 100 mL) equipped with a stir bar was sequentially charged with **Monomer I** (9.0 mmol, 0.90 equiv) and **Monomer II** (1.0 mmol, 0.10 equiv). The reaction flask was evacuated and backfilled with nitrogen from the Schlenk line (this process was repeated a total of three times), then anhydrous DMF (12 mL) were added successively. The reaction mixture was stirred at room temperature for 5 min and then 4,4′-(hexafluoroisopropylidene)diphthalic anhydride (**6FDA**) (4.44 g, 10 mmol. 1.0 equiv) was added. The solution was stirred at room temperature for 24-48 hours to form polyamic acid. Next, a solution of triethylamine (1.4 mL, 10 mmol, 1.0 equiv) and acetic anhydride (3.8 mL, 40 mmol, 4.0 equiv) dissolved in anhydrous DMF (2 mL) was added. The mixture was vigorously stirred for 20 hours to allow complete imidization. ***Workup***: The obtained mixture was diluted by adding 10 mL DMF, and then dropped slowly into water to obtain white polyimide fibers or balls. The collected polymer was washed several times by water before drying in a vacuum oven at 150 ^o^C for 48 h.

# Characterization Data for Polymers

## 3.1 Characterization Data for Melamine-Based Copolyimides

**6FDA-DAM:MEL-I 9:1 (P1):** The general procedure was followed on a 10 mmol scale using **6FDA** (4.44 g, 10 mmol, 1.0 equiv), 2,4,6-Trimethyl-m-phenylenediamine (**DAM**) (1.35 g, 9.0 mmol, 0.90 equiv), and 2,4-Diamino-6-[3-(trifluoromethyl)phenyl]-1,3,5-triazine (**MEL-I)** (255 mg, 1.0 mmol, 0.10 equiv). The reaction mixture was stirred at room temperature for 72 h to form polyamic acid. After ***Workup***, the title polyimide was obtained as white balls (5.41 g, 95% yield). *6FDA-DAM part*: **^1^H NMR** (500 MHz, DMSO-d_6_) δ 8.25 – 8.10 (m, 2H), 7.92 (s, 4H), 7.32 (s, 0.9H), 2.14 (s, 5.4H), 1.92 (s, 2.7H). *Melamine part*: δ 8.57 (s, 0.1H), 8.52 (d, *J* = 7.9 Hz, 0.1H), 8.29 (d, *J* = 8.2 Hz, 0.1H), 7.76 – 7.70 (m, 0.1H) **^19^F NMR** (471 MHz, DMSO-d_6_) δ -61.2, -62.8. Based on ^1^H-NMR, the ratio of **DAM** to **MEL-I** is 9:1. This ratio indicated that on average, 0.9 **DAM** and 0.1 **MEL-I** units were incorporated on each repeat unit of **P1**. **IR** (neat, cm^−1^) 2928, 2857, 2366, 2342, 2321, 1787, 1730, 1542, 1488, 1436, 1359, 1298, 1256, 1210, 1193, 1143, 1106, 1039, 983, 962, 863, 750, 724, 705, 627, 545. Molecular weight values were calculated using ChemStation GPC Data Analysis Software (Rev. B.01.01) based on the refractive index signal. *M*n = 21.7 kg/mol, *M*w = 36.6 kg/mol, PDI = 1.7 (Figure S1a).

**6FDA-DAM:MEL-II 9:1 (P2):** The general procedure was followed on a 10 mmol scale using **6FDA** (4.44 g, 10 mmol, 1.0 equiv), **DAM** (1.35 g, 9.0 mmol, 0.90 equiv), and 2-morpholine-4,6-diamino-1,3,5-triazine (**MEL-II**) (196 mg, 1.0 mmol, 0.10 equiv). The reaction mixture was stirred at room temperature for 72 h to form polyamic acid. After ***Workup***, the title polyimide was obtained as white balls (5.35 g, 95% yield). *6FDA-DAM part*: **^1^H NMR** (500 MHz, DMSO-d_6_) δ 8.32 – 8.12 (m, 2H), 8.05 – 7.83 (m, 4H), 7.32 (s, 0.9H), 2.14 (s, 5.4H), 1.91 (s, 2.7H). *Melamine part*: δ 3.76 – 3.51 (m, 0.8H). **^19^F NMR** (471 MHz, THF-d_8_) δ -63.9. Based on ^1^H-NMR, the ratio of **DAM** to **MEL-II** is 9:1. This ratio indicated that on average, 0.9 **DAM** and 0.1 **MEL-II** units were incorporated on each repeat unit of **P2**. **IR** (neat, cm^−1^) 2925, 2861, 2361, 2343, 1792, 1731, 1654, 1560, 1508, 1489, 1358, 1297, 1256, 1210, 1143, 1105, 982, 863, 724, 739, 705, 543. Molecular weight values were calculated using ChemStation GPC Data Analysis Software (Rev. B.01.01) based on the refractive index signal. *M*n = 14.1 kg/mol, *M*w = 27.1 kg/mol, PDI = 1.9 (Figure S1b).

**6FDA-6FpDA:MEL-I 9:1 (P3):** The general procedure was followed on a 8.0 mmol scale using **6FDA** (3.55 g, 8.0 mmol, 1.0 equiv), 4,4′-(hexafluoroisopropylidene)dianiline (**6FpDA**) (2.41 g, 7.2 mmol, 0.90 equiv), **MEL-I** (204 mg, 0.80 mmol, 0.10 equiv), and DMF (10 mL). The reaction mixture was stirred at room temperature for 24 h to form polyamic acid. After ***Workup***, the title polyimide was obtained as white balls (5.76 g, 98% yield). *6FDA-6FpDA part*: **^1^H NMR** (500 MHz, THF-d_8_) *6FDA-6FpDA part*: δ 8.11 (d, *J* = 8.0 Hz, 2H), 8.00 (d, *J* = 8.1 Hz, 2H), 7.90 (s, 2H), 7.71 – 7.55 (m, 7.2H). *Melamine part*: δ 8.70 (s, 0.1H), 8.59 (d, *J* = 7.8 Hz, 0.1H), 8.23 – 8.16 (m, 0.1H), 7.75 (d, *J* = 7.8 Hz, 0.1H). **^19^F NMR** (471 MHz, THF-d_8_) δ -63.3, -64.0, -64.2. Based on ^1^H-NMR, the ratio of **6FpDA** to **MEL-I** is 9:1. This ratio indicated that on average, 0.9 **6FpDA** and 0.1 **MEL-I** units were incorporated on each repeat unit of **P3**. **IR** (neat, cm^−1^) 2967, 2925, 2372, 2349, 2340, 1725, 1560, 1541, 1518, 1508, 1374, 1256, 1208, 1177, 1150, 1103, 988, 901, 854, 828, 718, 644, 594, 529. Molecular weight values were calculated using ChemStation GPC Data Analysis Software (Rev. B.01.01) based on the refractive index signal. *M*n = 21.5 kg/mol, *M*w = 39.4 kg/mol, PDI = 1.8 (Figure S1c).

**6FDA-6FpDA:MEL-I 4:1 (P4):** The general procedure was followed on a 8.0 mmol scale using **6FDA** (3.55 g, 8.0 mmol, 1.0 equiv), **6FpDA** (2.14 g, 6.4 mmol, 0.80 equiv), **MEL-I** (408 mg, 1.6 mmol, 0.20 equiv), and DMF (10 mL). The reaction mixture was stirred at room temperature for 48 h to form polyamic acid. After ***Workup***, the title polyimide was obtained as white balls (5.58 g, 96% yield). **^1^H NMR** (500 MHz, THF-d_8_) *6FDA-6FpDA part*: δ 8.12 (d, *J* = 8.0 Hz, 2H), 8.01 (d, *J* = 8.1 Hz, 2H), 7.91 (s, 2H), 7.71 – 7.55 (m, 6.4H). *Melamine part*: δ 8.70 (s, 0.2H), 8.59 (d, *J* = 7.8 Hz, 0.2H), 8.23 – 8.16 (m, 0.2H), 7.75 (d, *J* = 7.8 Hz, 0.2H). **^19^F NMR** (471 MHz, THF-d_8_) δ -63.3, -64.0, -64.2. Based on ^1^H-NMR, the ratio of **6FpDA** to **MEL-I** is 4:1. This ratio indicated that on average, 0.8 **6FpDA** and 0.2 **MEL-I** units were incorporated on each repeat unit of **P4**. **IR** (neat, cm^−1^) 3015, 2992, 2975, 2953, 2361, 2342, 1792, 1727, 1521, 1412, 1398, 1373, 1256, 1208, 1177, 1139, 944, 854, 828, 718, 594, 536. Molecular weight values were calculated using ChemStation GPC Data Analysis Software (Rev. B.01.01) based on the refractive index signal. *M*n = 18.0 kg/mol, *M*w = 27.3 kg/mol, PDI = 1.5 (Figure S1d).

## 3.2 Characterization Data for Monomers

**2-morpholine-4,6-diamino-1,3,5-triazine (MEL-II):** 2-chloro-4.6-diamino-1,3,5-triazine (10.0 g, 69 mmol, 1.0 equiv, 90% purity), morpholine (12.0 mL, 138 mmol, 2.0 equiv), K_2_CO_3_ (19.0 g, 138 mmol, 2.0 equiv), and DMF (50mL) were added to a 250 mL flask equipped with a stir bar. The reaction mixture was stirred at 100 ^o^C for 18 h. The reaction mixture was cooled down to room temperature and transferred to a 500 mL beaker. 200 mL saline water was added to the mixture and the mixture was cooled to 10 ^o^C. The precipitated crystal was separated by filtration. The collected product was washed several times by water and acetonitrile before drying in a vacuum oven at 100 ^o^C overnight. The product was obtained as a white solid (8.3 g, 69% yield). **m.p.** 245–255 °C. **^1^H NMR** (500 MHz, DMSO-d_6_) δ 6.15 (s, 4H), 3.61 – 3.58 (m, 4H), 3.57 – 3.54 (m, 4H). **^13^C NMR** (126 MHz, DMSO-d_6_) δ 167.2, 165.5, 66.1, 43.1. **IR** (neat, cm^-1^) 3299, 3155, 1621, 1537, 1479, 1433, 1369, 1306, 1292, 1130, 1112, 1102, 1065, 998, 883, 810, 616, 582, 538.

# NMR Spectra

**^1^H NMR** (500 MHz, DMSO-d_6_)

**^19^F NMR** (471 MHz, DMSO-d_6_)

**^1^H NMR** (500 MHz, DMSO-d_6_)

**^19^F NMR** (471 MHz, THF-d_8_)

**^1^H NMR** (500 MHz, THF-d_8_)

**^19^F NMR** (471 MHz, THF-d_8_)

**^1^H NMR** (500 MHz, THF-d_8_)

**^19^F NMR** (471 MHz, THF-d_8_)

**^1^H NMR** (500 MHz, DMSO-d_6_)

**^13^C NMR** (126 MHz, DMSO-d_6_)

# Gel Permeation Chromatography

**Figure S1a.** GPC trace of **P1.**

**Figure S1b.** GPC trace of **P2.**

**Figure S1c.** GPC trace of **P3.**

**Figure S1d.** GPC trace of **P4.**

# Thermogravimetric Analysis and Differential Scanning Calorimetry

| **Polymer** | ${\boldsymbol{T}_{\boldsymbol{G}}\mathbf{(}}^{\boldsymbol{o}}\boldsymbol{C}\mathbf{)}$ | ${\boldsymbol{T}_{\boldsymbol{D}}\mathbf{(}}^{\boldsymbol{o}}\boldsymbol{C}\mathbf{)}$ |
| --- | --- | --- |
| **6FDA-DAM: MEL-I 9:1 (P1)** | 353.3 | 525.9 |
| **6FDA-DAM: MEL-II 9:1 (P2)** | 354.4 | 518.5 |
| **6FDA-6FpDA: MEL-I 9:1 (P3)** | 292.8 | 512.8 |
| **6FDA-6FpDA: MEL-I 4:1 (P4)** | 268.5 | 508.8 |

**Table S1.** Glass transition temperature ($T_{G}$) and degradation temperature ($T_{D}$) of all four polymers, 6FDA-DAM: MEL-I 9:1 (P1), 6FDA-DAM: MEL-II 9:1 (P2), 6FDA-6FpDA: MEL-I 9:1 (P3), 6FDA-6FpDA: MEL-I 4:1 (P4)


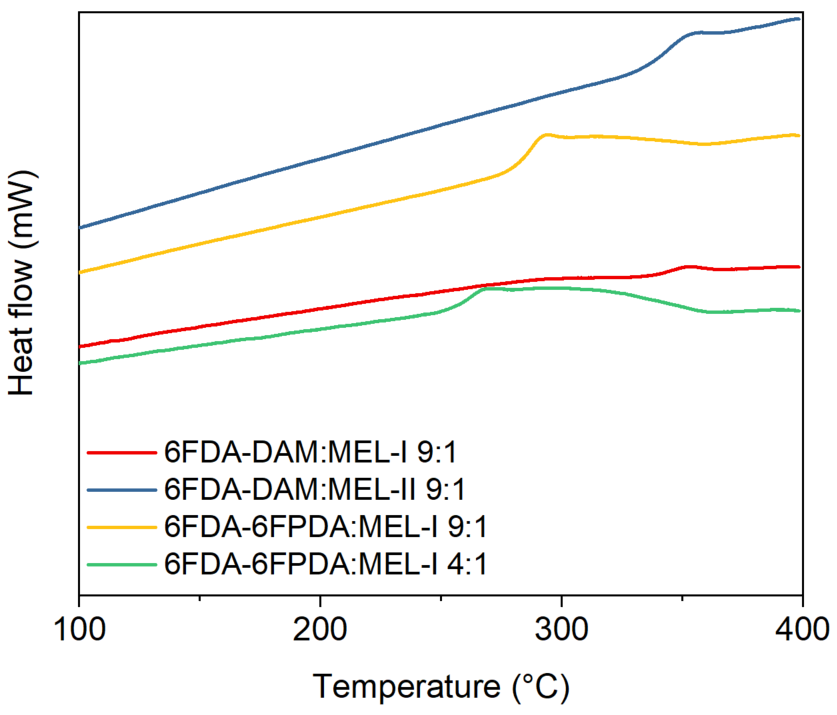


**Figure S2-1.** Differential Scanning Calorimetry (DSC) scans for **P1**, **P2**, **P3**, **P4**.


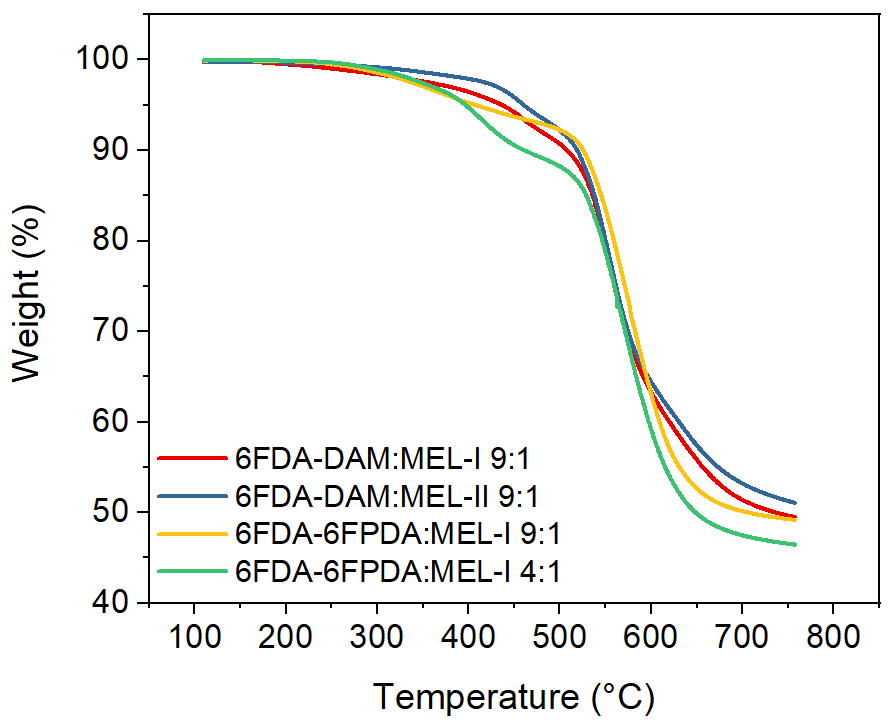


**Figure S2-2.** Thermogravimetric Analysis (TGA) scans for **P1**, **P2**, **P3, P4.**

# Membrane Fabrication

The co-polyimide dense film membrane was prepared by a solution casting method. 4 grams of vacuum dried polymer (6FDA-DAM: MEL-I 9:1 (P1), 6FDA-DAM: MEL-II 9:1 (P2), 6FDA-6FpDA: MEL-I 9:1 (P3) or 6FDA-6FpDA: MEL-I 4:1 (P4)) were dissolved in 6 ml of tetrahydrofuran (THF) and sonicated for 20 minutes until no solid dispersion was observed. The homogenous dope solution was then filtered through a 0.2 μm PTFE filter. The low resistance during the filtering process indicated the high solubility of the polymers in common organic solvent such as THF. The homogeneous solution was then poured into flat-bottomed PTFE Petri dishes and covered with glass plates to prevent contamination from dust and control the solvent evaporation rate. The solutions were left to evaporate at ambient temperature and pressure in the fume hood for 48 hours to form free-standing films. Films were carefully peeled from the Petri dishes. Finally, the film was heated in vacuum oven to 150°C to remove any residual solvent, and then cooled slowly to room temperature.

All four polymer membrane films showed light transparent yellow color with good mechanical strength and high flexibility.


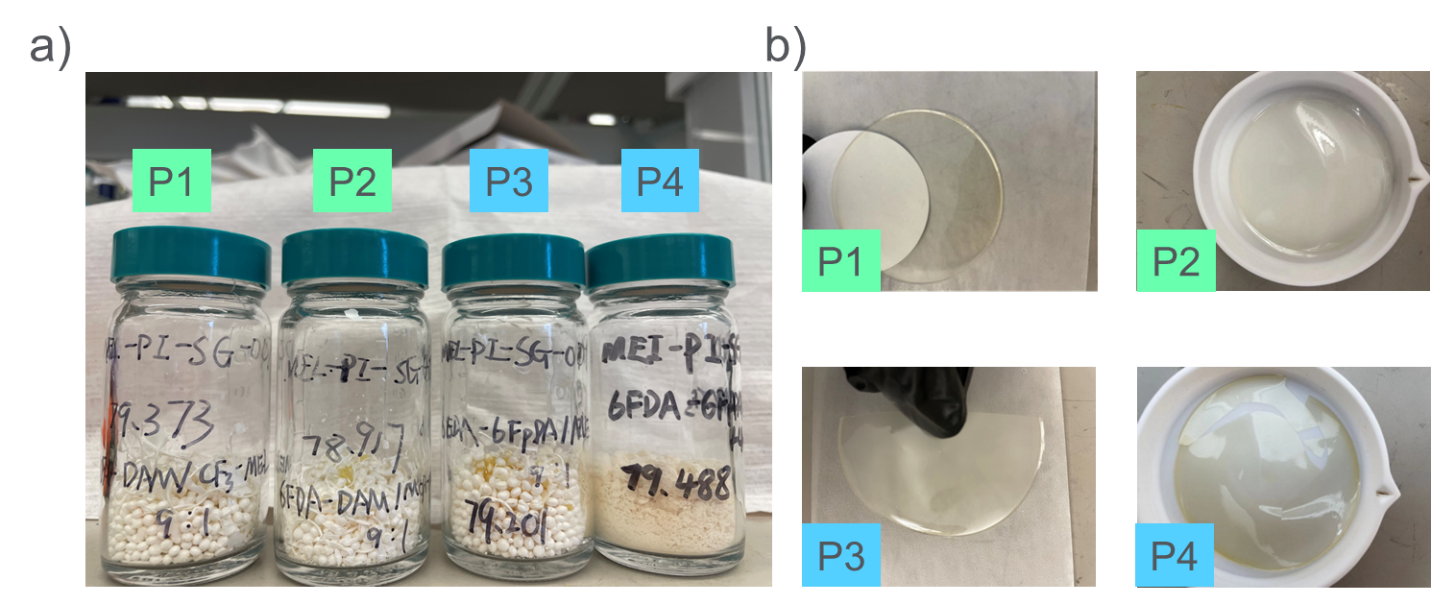


**Figure S3.** a) All four co-polyimide in powder form that is successfully synthesized. b) All four co-polyimide that is dissolved in homogenous solution and casted into dense film membranes.

# Membrane Permeation Testing

Gas permeation tests were performed in triplicate using a constant-volume, variable pressure technique. A schematic diagram of this custom-built permeation apparatus is shown in Figure S4. A stainless-steel permeation cell with 47 mm disc filters was purchased from EMD Milipore. An epoxy masked membrane of 5-20 mm in diameter was inserted and sealed in the testing cell, and the permeation system was completed evacuated overnight before each test. Pure gas permeability coefficients were measured at 25°C and feed pressure of 100 psi in order of CH_4_ followed by CO_2_ to avoid plasticization. Steady-state permeation was verified using the time-lag method, where 10 times the diffusion time-lag was taken as the effective steady-state. The upstream (feed) pressure and the downstream (permeate) pressure were measured using Baraton absolute capacitance transducers (MKS Instruments) and recorded using LabVIEW software. The permeate pressure was maintained below 10 torr. Mixed gas permeation was performed at 25°C and feed pressure range of 100 psi to 800 psi with binary gas mixture and sour gas mixtures. A retentate stream was added for mixed gas tests and adjusted to 100 times the permeate flow rate to maintain less than 1% stage cut. The permeate gas was collected and then injected into a Shimadzu gas chromatograph (GC-2014) to measure permeate composition. Permeate injections were performed at 15 torr. An Isco pump (Teledynelsco) was used to control the feed pressure.


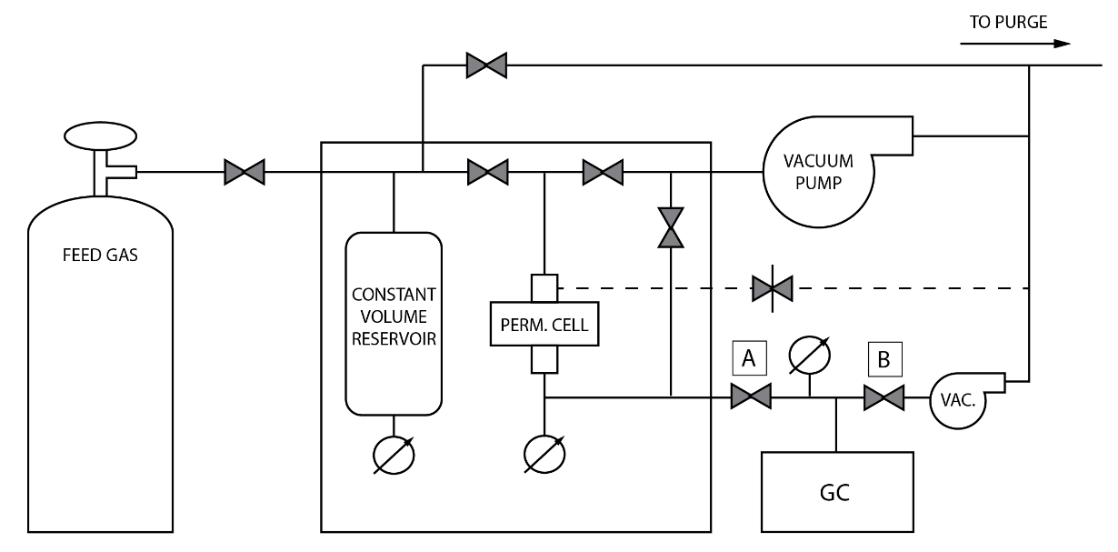


**Figure S4.** Schematic diagram of constant-volume, variable pressure permeation apparatus used for measuring single gas and mixed permeation properties.

Permeability coefficients of gas $i$, $P_{i}$ was calculated according to Equation S1, where $dp_{i}/dt$ is the slope of the steady state pressure rise in the downstream, $V$ is the downstream volume, $R$ is the ideal gas constant, $T$ is the temperature of the downstream, $L$ is the membrane thickness, A is the membrane surface area (estimated using ImageJ image processing software), and $\Delta f_{i}$ is the partial fugacity difference across the membrane calculated using the Peng-Robinson equation. Permselectivity, $\alpha_{i/j}$ was calculated as the ratio of permeability coefficients as expressed in Equation S2.

| $P_{i}=\frac{dp_{i}}{dt} \frac{V L}{R T A \Delta f_{i}}$ | (S1) |
| --- | --- |
| $\alpha_{i/j}=P_{i}/P_{j}$ | (S2) |

# Pure Gas Permeability Data

**Table S2.** Permeability data for P1, P2, P3 and P4 films.

| Polymer | Thickness (μm) | Treatment | Days Aged | $P_{{CO}_{2}}$ (barrer) | $P_{{CH}_{4}}$ (barrer) | $P_{N_{2}}$ (barrer) | $\alpha_{{CO}_{2}/{CH}_{4}}$ |
| --- | --- | --- | --- | --- | --- | --- | --- |
| P1 | 122 | 150°C under vacuum | 2 | 118.3$\pm$24.3 | 3.1$\pm$0.7 | 5.3$\pm$1.2 | 39.0$\pm1.6$ |
|  | 185 |  | 2 |  |  |  |  |
|  | 112 |  | 2 |  |  |  |  |
|  | 103 |  | 2 |  |  |  |  |
| P2 | 103 | 150°C under vacuum | 3 | 121.7$\pm$19.8 | 3.5$\pm0.4$ | 5.6$\pm$0.6 | 34.3$\pm1.0$ |
|  | 97 |  | 3 |  |  |  |  |
|  | 122 |  | 3 |  |  |  |  |
|  | 120 |  | 3 |  |  |  |  |
| P3 | 81 | 150°C under vacuum | 4 | 19.5$\pm$0.3 | 0.32$\pm$0.01 | 0.8$\pm$0.02 | 60.5$\pm$2.9 |
|  | 79 |  | 4 |  |  |  |  |
|  | 85 |  | 4 |  |  |  |  |
|  | 82 |  | 4 |  |  |  |  |
| P4 | 131 | 150°C under vacuum | 2 | 11.9$\pm$5.6 | 0.13$\pm$0.07 | 0.72$\pm0.022$ | 87.2$\pm3.7$ |
|  | 144 |  | 2 |  |  |  |  |
|  | 89 |  | 2 |  |  |  |  |

# Sorption Analysis

The sorption coefficient describes the thermodynamic contribution of a gas molecule transporting through a membrane and can be described as shown in Equation S3.

| $S_{i}=\frac{c_{i}}{f_{i}}$ | (S3) |
| --- | --- |

Where $c_{i}$ is the concentration of the sorbed gas species in the film at equilibrium and $f_{i}$ is the fugacity of the gas that the film membrane is exposed to during the sorption process. The sorption isotherms of 6FDA-DAM, P1, 6FDA-6FpDA, and P3 for CO_2_ and CH_4_ were measured using pressure decay sorption setup at 25 °C and are shown in Figure S5.


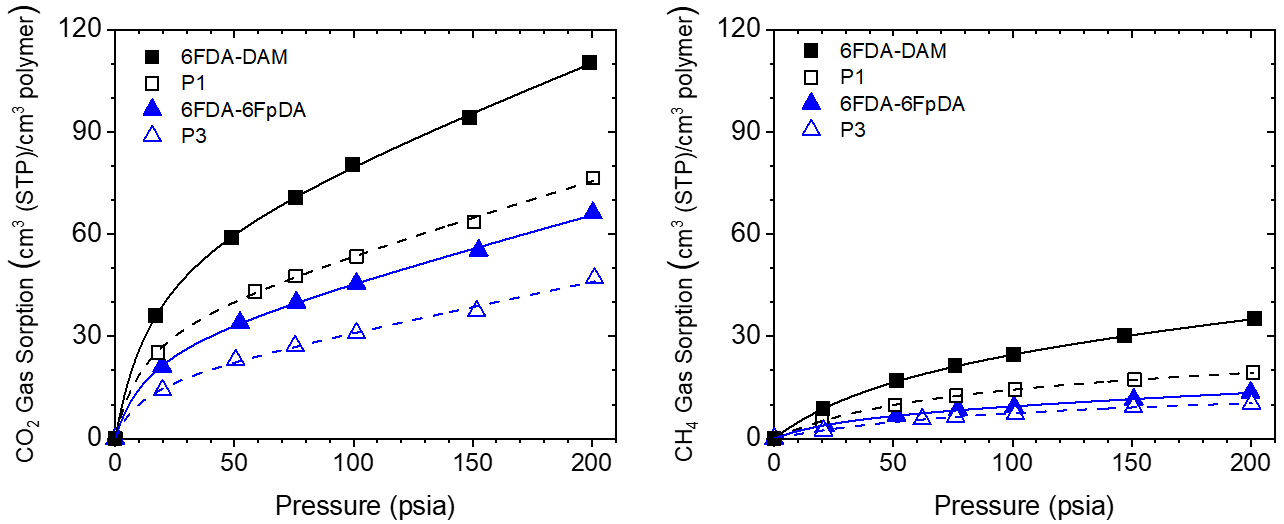


**Figure S5.** CO_2_ and CH_4_ sorption isotherm for four polymers, 6FDA-DAM, P1, 6FDA-6FpDA, P3 with pressure up to 200 psia at 25 °C.

With the isotherm, the CO_2_ and CH_4_ solubility and diffusion coefficient at 100 psi can be obtained as shown in Table S3.

**Table S3.** CO_2_ and CH_4_ solubility and diffusivity data for 6FDA-DAM, P1, 6FDA-6FpDA, P3

| Membrane | Solubility  (X 10^2^ cm^3^ (STP)/(cm^3^・cmHg)) | | Diffusivity  (X 10^8^ cm^2^/s) | | Sorption selectivity | Diffusion selectivity |
| --- | --- | --- | --- | --- | --- | --- |
|  | CO_2_ | CH_4_ | CO_2_ | CH_4_ | CO_2_/CH_4_ | CO_2_/CH_4_ |
| 6FDA-DAM | 15.56 | 4.71 | 34.87 | 5.54 | 3.30 | 6.29 |
| P1 | 10.20 | 2.75 | 11.61 | 1.10 | 3.71 | 10.55 |
| 6FDA-6FpDA | 8.69 | 1.79 | 7.65 | 0.84 | 4.85 | 9.11 |
| P3 | 5.92 | 1.38 | 3.30 | 0.234 | 4.29 | 14.10 |

For glassy polymers, the sorption relationship is usually described by the dual-mode sorption model as shown in Equation S4.

| $c_{i}=c_{D,i}+c_{H,i}=k_{D,i}\cdot f_{i}+\frac{C_{H,i}^{'}\cdot b_{i}\cdot f_{i}}{1+b_{i}\cdot f_{i}}$ | (S4) |
| --- | --- |

Where $c_{D,i}$ and $c_{H,i}$ are the gas concentration sorbed in Henry’s law and Langmuir environments, respectively. In Equation S4, $k_{D,i}$ is the Henry’s law sorption coefficient, $C_{H,i}^{'}$ is the Langmuir capacity constant, and $b_{i}$ is the Langmuir affinity constant. Equation S4 can be further extended to account for binary gas mixtures as shown in equation S5 and S6.

| $c_{A}=k_{D,A}\cdot f_{A}+\frac{C_{H,i}^{'}\cdot b_{A}\cdot f_{A}}{1+b_{A}\cdot f_{A}+b_{B}\cdot f_{B}}$ | (S5) |
| --- | --- |
| $c_{B}=k_{D,B}\cdot f_{B}+\frac{C_{H,i}^{'}\cdot b_{B}\cdot f_{B}}{1+b_{A}\cdot f_{A}+b_{B}\cdot f_{B}}$ | (S6) |

In this paper, Equation S5 and S6 will be used to predict CH_4_ and CO_2_ sorption isotherms in 20/80 binary CH_4_ and CO_2_ mixture. The dual mode sorption parameters ($k_{D,i}, C_{H,i}^{'}$ and $b_{i}$) for all polymers used for mixture gas sorption prediction are obtained through fitting the pure CH_4_ and CO_2_ isotherms (Figure S5) using equation S4. The obtained dual mode sorption parameters are shown in Table S4. The predicted mixture sorption isotherm is shown in Figure S6 using Table S4 and equation S5-S6.

**Table S4.** Dual mode parameters for 6FDA-DAM, P1, 6FDA-6FpDA, P3

| Membrane | CH_4_ | | | CO_2_ | | |
| --- | --- | --- | --- | --- | --- | --- |
|  | $k_{D}$ (cc STP / (cc polymer • psi)) | $b$ (1/psia) | $C_{H}^{'}$ (cc STP / cc polymer) | $k_{D}$ (cc STP / (cc polymer • psi)) | $b$ (1/psia) | $C_{H}^{'}$ (cc STP / cc polymer) |
| 6FDA-DAM | 0.0592 | 0.0162 | 30.275 | 0.265 | 0.0609 | 61.685 |
| P1 | 0.0177 | 0.0147 | 21.161 | 0.202 | 0.0805 | 37.316 |
| 6FDA-6FpDA | 0.03 | 0.0285 | 8.714 | 0.185 | 0.0712 | 30.404 |
| P3 | 0.0059 | 0.01 | 13.875 | 0.141 | 0.0855 | 18.768 |


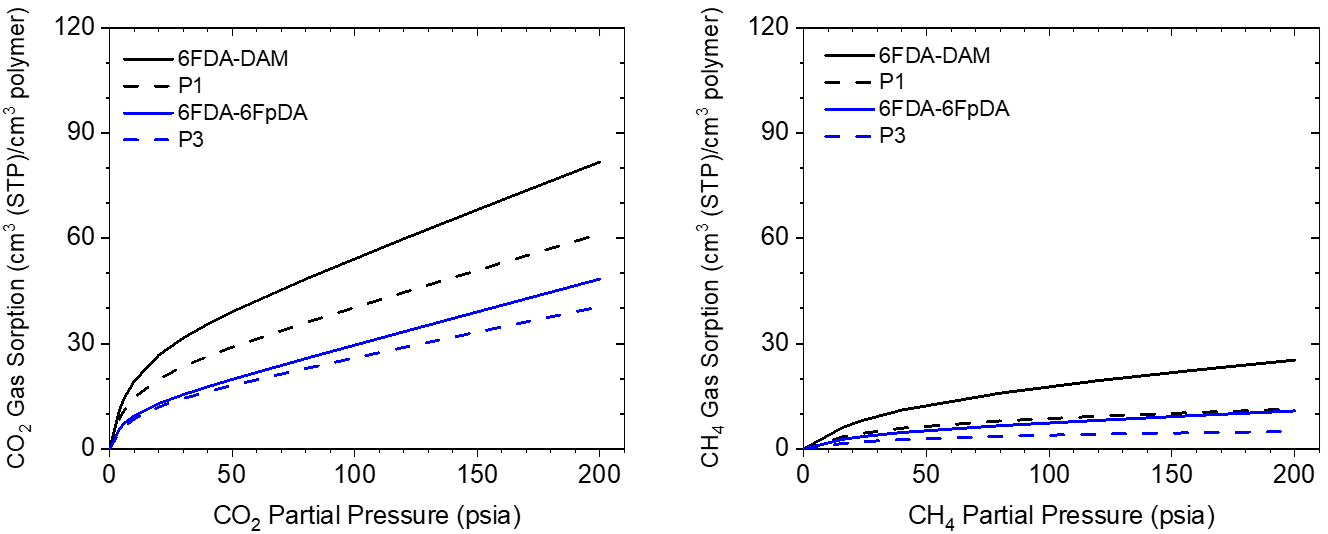


**Figure S6.** 20 mol% CO_2_/ 80 mol% CH_4_ mixture sorption isotherm from dual mode sorption prediction for four polymers, 6FDA-DAM, P1, 6FDA-6FpDA, P3 with pressure up to 200 psia of partial pressure at 25 °C.

The biggest “upgrade” comparing P1 and P3 with their pristine polymers, 6FDA-DAM and 6FDA-6FpDA is the increase in CO_2_ $b$ value, which is the CO_2_ Langmuir affinity constant, making P1 and P3 having significant enhancement in binary mixture selectivity due to competitive sorption.

# Binary Gas Permeability Data

**Table S5.** CO_2_/CH_4_ mixed-gas permeability data for 6FDA-DAM: MEL-I 9:1 (P1) and 6FDA-6FpDA: MEL-I 9:1 (P3)

| **Membrane** | **Mixture Composition** | **Feed Pressure (psi)** | $\boldsymbol{P}_{\boldsymbol{CO}_{\boldsymbol{2}}}$ **(barrer)** | $\boldsymbol{P}_{\boldsymbol{CH}_{\boldsymbol{4}}}$**(barrer)** | $\boldsymbol{\alpha}_{\boldsymbol{CO}_{\boldsymbol{2}}\boldsymbol{/}\boldsymbol{CH}_{\boldsymbol{4}}}$ |
| --- | --- | --- | --- | --- | --- |
| **P1** | 20:80 CO_2_ /CH_4_ | 100 | 113±1.6 | 1.80±0.1 | 62 |
|  | 20:80 CO_2_ /CH_4_ | 200 | 106±1.3 | 2.06±0.04 | 51 |
|  | 20:80 CO_2_ /CH_4_ | 500 | 93±1.5 | 2.02±0.03 | 46 |
|  | 20:80 CO_2_ /CH_4_ | 800 | 87±1.3 | 2.06±0.03 | 42 |
| **P3** | 20:80 CO_2_ /CH_4_ | 200 | 18.8±0.2 | 0.17±0.002 | 107 |
|  | 20:80 CO_2_ /CH_4_ | 500 | 16.1±0.2 | 0.17±0.002 | 94 |
|  | 20:80 CO_2_ /CH_4_ | 800 | 15.0±0.1 | 0.20±0.002 | 74 |

# Business case sour natural gas with five components

**Table S6.** Separation performance for 6FDA-6FpDA-MEL-I 9:1 (P3) using business case five component sour natural gas feed with 10 mol% CO_2_, 20 mol% H_2_S, 57 mol% CH_4_, 3 mol% C_2_H_6_ and 10 mol% N_2_ under 25°C. All permeability $P$ is shown in barrer.

| **Pressure (psi)** | $\boldsymbol{P}_{\boldsymbol{CO}_{\boldsymbol{2}}}$ | $\boldsymbol{P}_{\boldsymbol{H}_{\boldsymbol{2}}\boldsymbol{S}}$ | $\boldsymbol{P}_{\boldsymbol{C}_{\boldsymbol{2}}\boldsymbol{H}_{\boldsymbol{6}}}$ | $\boldsymbol{P}_{\boldsymbol{N}_{\boldsymbol{2}}}$ | $\boldsymbol{P}_{\boldsymbol{CH}_{\boldsymbol{4}}}$ | $\boldsymbol{\alpha}_{\boldsymbol{CO}_{\boldsymbol{2}}\boldsymbol{/}\boldsymbol{CH}_{\boldsymbol{4}}}$ | $\boldsymbol{\alpha}_{\boldsymbol{H}_{\boldsymbol{2}}\boldsymbol{S}\boldsymbol{/}\boldsymbol{CH}_{\boldsymbol{4}}}$ |
| --- | --- | --- | --- | --- | --- | --- | --- |
| 280 | 14.72±0.0 | 0.93±0.02 | 0.04±0.01 | 3.17±0.01 | 0.19±0.0 | 77±0.6 | 4.9±0.1 |
| 500 | 14.87±0.05 | 2.20±0.06 | 0.04±0.01 | 1.32±0.00 | 0.20±0.00 | 73±1.5 | 10.8±0.0 |
| 700 | 15.04±0.15 | 2.32±0.02 | 0.05±0.00 | 1.36±0.03 | 0.21±0.01 | 72±4.6 | 11.05±0.5 |

# Sour gas mixed gas separation performance

Table S7. Comparison of glassy polymer membrane performance on H_2_S/CO_2_/CH_4_ separation

| Polymers | Composition  (H_2_S/CO_2_/CH_4_/N_2_/C_2_) | Temp. (°C) | Feed (bar) | Permeability (Barrer) | | | Selectivity | | | Ref |
| --- | --- | --- | --- | --- | --- | --- | --- | --- | --- | --- |
|  |  |  |  | H_2_S | CO_2_ | H_2_S+CO_2_ | H_2_S/CH_4_ | CO_2_/CH_4_ | (H_2_S+CO_2_)/CH_4_ |  |
| 6FDA-DAM:DABA 3:2 | 25/5/70 | 35 | 48.3 | 106.7 | 97.73 | 204.4 | 24.49 | 22.43 | 46.92 | ^[1]^ |
| 6FDA-DAM aged | 20/20/60 | 35 | 46 | 495.1 | 301.1 | 796.1 | 31.2 | 19 | 50.2 | ^[2]^ |
| 6FDA-mPDA-(6FDA-durene) | 20/10/60/10 | 22 | 34.5 | 11 | 13 | 26 | 23 | 27 | 50 | ^[2]^ |
| 6FDA-6FpDA/6FDA-Durene(4:1) | 20/10/59/10/1 | 22 | 34.5 | 26.4 | 41.7 | 68.1 | 13.1 | 20.8 | 33.9 | ^[3]^ |
| 6FDA-TPA | 20/10/59/10/1 | 22 | 34.5 | 67.08 | 50.12 | 117.2 | 17.8 | 13.3 | 31.1 | ^[4]^ |
| 6F-PAI-2 | 20/20/60 | 35 | 55.2 | 4 | 6.5 | 10.5 | 6.5 | 12.5 | 19 | ^[2]^ |
| 6FDA-TPA (t-Bu) | 20/10/59/10/1 | 22 | 34.6 | 66.02 | 50.04 | 116.06 | 15 | 11.4 | 26.4 | ^[4]^ |
| 6FDA-DAM:DABA 1:1 | 25/5/70 | 35 | 48.3 | 27.87 | 37.99 | 65.86 | 22.65 | 26.38 | 49.03 | ^[1]^ |
| 6FDA-DAM:DABA 1:2 | 25/5/70 | 35 | 48.3 | 12.18 | 19.82 | 32.0 | 20.71 | 33.7 | 54.41 | ^[1]^ |
| CA | 20/20/60 | 35 | 48.3 | 39.7 | 27.5 | 67.2 | 27.4 | 19.1 | 46.5 | ^[2]^ |
| GCV-CA | 20/20/60 | 35 | 48.3 | 109 | 110 | 220 | 27.4 | 19.1 | 46.5 | ^[2]^ |
| PIM-6FDA-OH | 15/15/70 | 35 | 48.3 | 63 | 52.6 | 115.6 | 30 | 25 | 55 | ^[2]^ |
| AO-PIM-1 aged | 20/20/60 | 35 | 55.2 | 500 | 140 | 640 | 61 | 15 | 76 | ^[2]^ |
| TEGMC | 20/20/60 | 35 | 48.3 | 40.5 | 50 | 90.5 | 23.5 | 29 | 52.5 | ^[2]^ |
| DEGMC | 20/20/60 | 35 | 55.2 | 38.2 | 54.6 | 92.8 | 19.3 | 28.4 | 47.7 | ^[2]^ |
| 6FDA-DAM:DABA 3:2 (180°C) | 10/20/70 | 35 | 62 | 25.4 | 55.6 | 81 | 14.7 | 32.1 | 46.8 | ^[2]^ |
| Matrimid | 5/3/92 | 35 | 55.2 | 4.42 | 4.96 | 9.39 | 34.1 | 38.1 | 72.2 | ^[2]^ |
| CA | 5/3/92 | 35 | 55.2 | 4.14 | 4.14 | 8.56 | 39.9 | 37.4 | 77.3 | ^[2]^ |
| CA | 6/29/65 | 35 | 10.1 | 2.13 | 2.43 | 4.56 | 19.0 | 22.0 | 41 | ^[5]^ |
| 6F-PAI-1 | 10/20/70 | 35 | 63.3 | 4.20 | 8.10 | 12.30 | 11.0 | 32.0 | 44 | ^[6]^ |
| 6FDA-DAM/6FpDA (1:3) | 20/10/57/10/3 | 22 | 34.5 | 68 | 84.3 | 152.3 | 19.7 | 24.4 | 44.1 | ^[7]^ |
| 6FDA-DAM/CARDO (1:3) | 20/10/57/10/3 | 22 | 34.5 | 70.9 | 56 | 126.9 | 23.4 | 18.4 | 41.8 | ^[7]^ |
| 6FDA-DAM/ABL-21 (1:3) | 20/10/57/10/3 | 22 | 34.5 | 32.9 | 53.9 | 86.8 | 15.9 | 26 | 41.9 | ^[7]^ |
| 6FDA-6FpDA-MEL-I 9:1 | 20/10/57/10/3 | 25 | 47.6 | 2.32 | 15.04 | 17.36 | 11.05 | 72.0 | 83.05 | this wk |

# References

[1] Z. Liu, Y. Liu, W. Qiu, W. J. Koros, *Angew. Chem. Int. Ed.* **2020**, *59*, 14877-14883.

[2] J. D. Wind, D. R. Paul, W. J. Koros, *J. Membr. Sci.* **2004**, *228*, 227-236.

[3] W. Qiu, L. Xu, C.-C. Chen, D. R. Paul, W. J. Koros, *Polymer* **2013**, *54*, 6226-6235.

[4] J. Vaughn, W. J. Koros, *Macromolecules* **2012**, *45*, 7036-7049.

[5] J. E. Bachman, J. R. Long, *Energy Environ. Sci.* **2016**, *9*, 2031-2036.

[6] J. D. Wind, C. Staudt-Bickel, D. R. Paul, W. J. Koros, *Ind. Eng. Chem. Res.* **2002**, *41*, 6139-6148.

[7] P. S. Tin, T. S. Chung, Y. Liu, R. Wang, S. L. Liu, K. P. Pramoda, *J. Membr. Sci.* **2003**, *225*, 77-90.
